# Tannin in Cholera

**Published:** 1851-10

**Authors:** 


*Tannin in Cholera.*—During the recent epidemic of cholera, tannin was recommended and employed by the younger Græfe of Berlin. He prescribed it in upwards of thirty cases, several of which were in a state of collapse, and of these two only proved fatal. The dose was from five to ten grains, half-hourly or hourly. Dr. Willebrand, of Finland, in reporting the treatment to the Medical Society of Sweden, states that he was himself a witness of this brilliant success.—*Lond. Lancet, from Förhandlinger vid Svenska Laakare-Saallskapets Sammanskomster.*

